# Enhancing gonorrhea surveillance in China by testing females in gynecology clinics: Lessons learned from a pilot survey

**DOI:** 10.1371/journal.pone.0238710

**Published:** 2020-09-10

**Authors:** Wen-Ge Li, Chang-Hui Cai, Xin-Chao Cai, Shao-Chun Chen, Stijn van der Veen, Yue-Ping Yin

**Affiliations:** 1 Zhongshan Affiliated Hospital of Sun Yat-sen University, Zhongshan, China; 2 Department of Outpatient, The Second People’s Hospital of Zhongshan City, Zhongshan, China; 3 Department of Outpatient, Zhongshan Hospital of Traditional Chinese Medicine, Zhongshan, China; 4 Institute of Dermatology, Chinese Academy of Medical Sciences & Peking Union Medical College, Nanjing, China; 5 National Center for STD Control, China Center for Disease Control and Prevention, Nanjing, China; 6 Department of Microbiology and Parasitology, School of Medicine, Zhejiang University, Hangzhou, China; 7 Department of Dermatology, Sir Run Run Shaw Hospital, School of Medicine, Zhejiang University, Hangzhou, China; 8 State Key Laboratory for Diagnosis and Treatment of Infectious Diseases, Collaborative Innovation Center for Diagnosis and Treatment of Infectious Diseases, The First Affiliated Hospital, School of Medicine, Zhejiang University, Hangzhou, China; Emory University School of Medicine, UNITED STATES

## Abstract

**Objectives:**

China has a high burden of gonorrhea, but an imbalanced male-to-female (M/F) ratio of reported cases. Therefore, the prevalence of gonorrhea in China may be underestimated due to inadequate testing of potentially infected females. The objective of this study is to investigate the cause of this imbalanced M/F ratio and develop strategies to enhance gonorrhea surveillance, particularly among females.

**Methods:**

The national center for STD control (NCSTDC) of China CDC collected data in Zhongshan city over the year 2018 from the National Notifiable Disease Report System (NNDRS) and the Hospital and Laboratory Information Systems (HIS and LIS) that obtains information from 24 hospitals.

**Results:**

Analysis of 1,542 reported cases of gonorrhea and the case distribution among different hospitals showed that most of the female cases (80.31%) were reported by gynecological clinics. The M/F ratio of reported cases varied between different hospitals and was dependent on the intensity of testing of females by their gynecological clinics.

**Conclusions:**

This study showed a significant correlation between M/F ratios and the relative contribution of female gonorrhea testing, especially in gynecology clinics. Enhancing gonorrhea testing among females should be advocated to improve surveillance in China.

## Introduction

China has a high burden of gonorrhea. In 2018, a total of 133,156 cases of gonorrhea were reported in China, yielding an incidence of 9.59 cases per 100,000 population, which is a 36% increase since 2014 [1]. However, while the contemporaneous male-to-female (M/F) ratio of reported gonorrhea cases in the USA is 1.46 [2], the reported gonorrhea cases in China show a large gender imbalance, with an average M/F ratio of 5.01 during the period 2014–2018. Therefore, the prevalence of gonorrhea among females might be underestimated in China, possibly due to insufficient testing of at risk female populations. To investigate whether the imbalanced reported M/F gonorrhea-case ratio reflects a realistic prevalence or whether it is actually related to insufficient female testing, the National Center for Sexually Transmitted Diseases Control (NCSTDC), an affiliate of the Chinese Center for Disease Control and Prevention, performed this pilot study in Zhongshan city (population of 1.769 million), one of its sentinel sites in southern China.

## Materials and methods

Data of reported gonorrhea cases in Zhongshan over the period January 1st to December 31st, 2018, was collected from National Notifiable Disease Report System (NNDRS). All the information was anonymized and could not identify individual participants during or after data collection. The reported cases were laboratory-confirmed according to national guidelines for diagnosis of gonorrhea in China.^3^ All cases were reported by clinical physicians and confirmed by public health staff in the hospitals or local CDC. Demographic and clinical information of the confirmed gonorrhea cases and total numbers tested patients were extracted from the Hospital and Laboratory Information Systems (HIS and LIS) covering 24 hospitals in Zhongshan. SPSS (version 20; IBM Corporation) was used to conduct all analyses including chi-square test and linear regression analysis.

The research project has been approved by the Medical Ethics Committee at the Institute of Dermatology, the Chinese Academy of Medical Sciences & Peking Union Medical College, and the National Center for Sexually Transmitted Disease Control (NCSTD) at Nanjing (approval number 2014-KY-026). The data were collected fully anonymous from the NNDRS and could not contact with patients. The need for consent was waived by the ethics committee.

## Results

**Fig 1:**
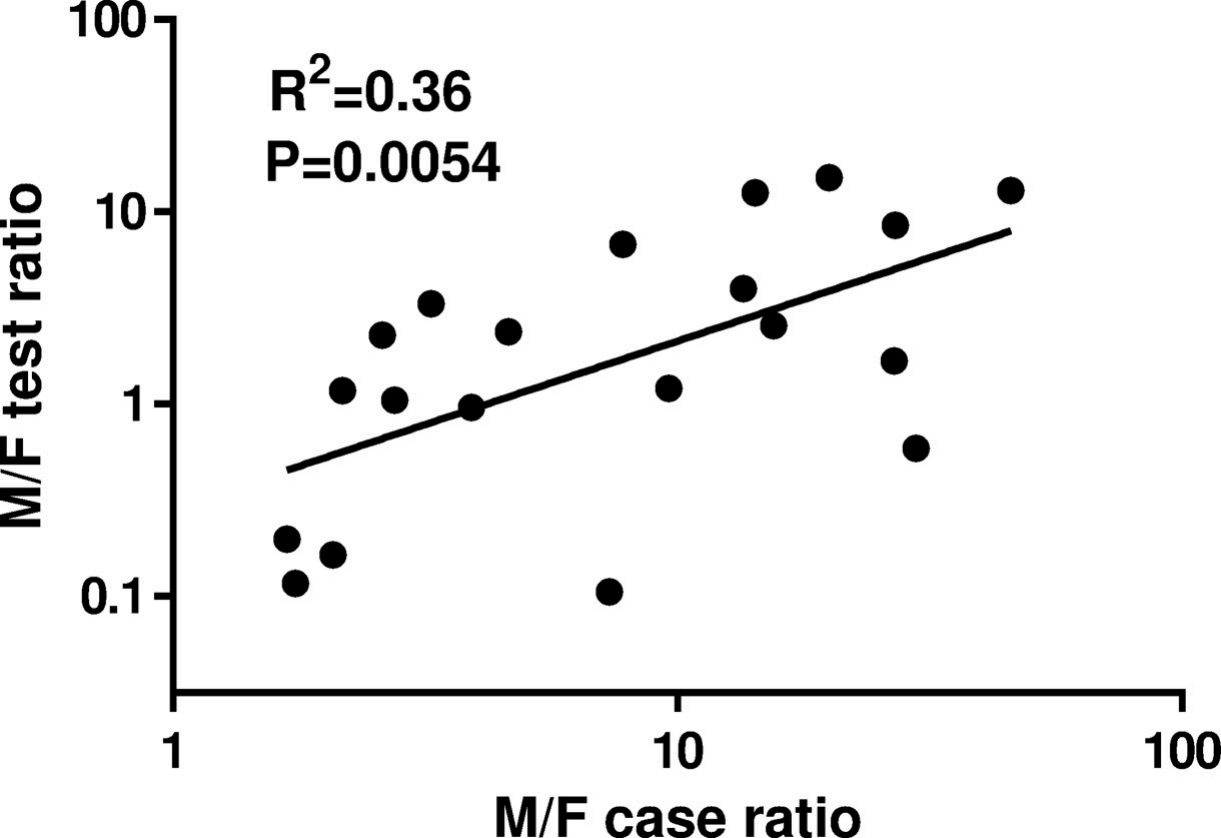
Correlation analysis between the male-to-female (M/F) gonorrhea case ratios and M/F gonorrhea testing ratios in 2018 among hospitals in Zhongshan city, China. Only 20 hospitals were included in the correlation analysis because 4 hospitals did not report any female gonorrhea cases.

A total of 13,308 patients (6,938 males and 6,370 females) were screened for gonorrhea in 2018 in 24 hospitals of Zhongshan city, resulting in the reporting of 1,542 laboratory-confirmed gonorrhea cases (positive rate: 12%) to NNDRS, corresponding to an incidence of 87.2 cases per 100,000 population. Among 1,542 positive cases, 6.5% (101 cases) were laboratory-confirmed by nucleic acid amplification test (NAAT) and 93.5% (1441 cases) were diagnosed by culture. The total number of male gonorrhea cases was 1,349 (73 were tested by NAAT and 1276 were tested by culture), which was much higher than the 193 female cases (28 were tested by NAAT and 165 were tested by culture), yielding an M/F case ratio of 6.99. However, the M/F case ratio varied among the 24 hospitals and ranged from 1.68 to 29.67 (median: 5.98), with 4 hospitals not reporting any female gonorrhea cases. To obtain more insight into the impact of female gonorrhea testing on M/F case ratios, the 24 hospitals were divided into three groups according to their M/F case ratios (group 1 ≤2; group 2 >2 and ≤5; group 3 >5) and the M/F ratios of the total number of tested patients among the three groups were subsequently analyzed. (Table 1) Indeed, the M/F test ratio of group 1 (0.17) was significantly lower compared with the M/F test ratios of group 2 (1.43) and group 3 (2.14), with half of all female gonorrhea testing being performed by the three hospitals included in group 1. Therefore, the M/F case ratios appeared to decrease in hospitals that perform more extensive female testing. This observation was further corroborated by linear regression analysis of log-normalized hospital M/F case and testing ratios, which showed a significant correlation between the relative contribution of female testing and M/F ratios among confirmed gonorrhea cases (Fig 1; P = 0.0054). These results indicate that the prevalence of gonorrhea among females is likely under-reported due to insufficient testing and that enhanced female testing for gonorrhea allows for the identification of more female gonorrhea cases, which will reduce the M/F case ratios.

**Table 1:** Reported gonorrhea cases, gonorrhea testing and clinic distribution in 2018 among 24 hospitals in Zhongshan city, China, grouped according to the hospital male-to-female (M/F) case ratios.

Groups§	Reported cases				Tested sample number and positivity				Clinic distribution of male reported gonorrhea cases			Clinic distribution of female reported gonorrhea cases			
	Male (NAAT/Culture)	Female (NAAT/Culture)	M/F Ratio	P	Numbers of tests among males (Positivity)	Numbers of tests among females (Positivity)	M/F Ratio	P	Urologic surgery clinic (%)	Dermatology and STD clinic (%)	Other clinics(%)	Gynecology clinic (%)	Dermatology and STD clinic (%)	Urologic surgery clinic (%)	Other clinics (%)
Group 1	147 (15/132)	81 (7/74)	1.81		528 (27.84%)	3163 (2.56%)	0.17		116 (78.91%)	29 (19.73%)	2 (1.36%)	74 (91.36%)	7 (8.64%)	0 (0.00%)	0 (0.00%)
Group 2	229 (56/173)	66 (14/52)	3.47	<0.001	926 (24.73%)	649 (10.17%)	1.43	<0.001	189 (82.53%)	37 (16.16%)	3 (1.31%)	50 (75.76%)	11 (16.67%)	3 (4.55%)	2 (3.03%)
Group 3	973 (2/971)	46 (7/39)	21.15	<0.001	5484 (17.74%)	2558 (1.80%)	2.14	<0.001	502 (51.59%)	428 (43.99%)	43 (4.42%)	31 (67.39%)	14 (30.43%)	0 (0.00%)	1 (2.17%)
Total	1349 (73/1276)	193 (28/165)	6.99		6938 (19.44%)	6370 (3.03%)	1.09		807 (59.82%)	494 (36.62%)	48 (3.56%)	155 (80.31%)	32 (16.58%)	3 (1.55%)	3 (1.55%)

^a^Groups: Group 1, M/F ratio ≤2 (3 hospitals); Group 2, M/F ratio > 2 and ≤5 (6 hospitals); Group 3, M/F ratio >5 (16 hospitals).

^b^chi-square test

The distribution of reported cases over the different clinics of the 24 hospitals was analysed based on clinical and laboratory information. Male cases were most commonly identified at the urologic surgery clinics (60%) and dermatology and STD clinics (37%), while female cases were mostly reported by gynecology clinics (80%) and dermatology and STD clinics (17%). (Table 1) Importantly, in China most of the female gonorrhea testing is done in the gynecology clinics, since 91% (5,771/6,370) of all female gonorrhea tests were prescribed by the gynecological clinics. Therefore, enhanced testing for gonorrhea in gynecology clinics should be an effective strategy to increase detection among females and improve gonorrhea surveillance.

Overall, enhancing of female testing for gonorrhea can be integrated into the national strategy of gonorrhea control and prevention in China. Female gonorrhea cases were mainly found in gynecology clinics, which suggests that these clinics should be the focus for improving surveillance among females in China.

## Discussion

This study found that strengthening the testing of female patients increases the identification of gonorrhea cases, which will improve gonorrhea surveillance and reduces further transmission of gonorrhea. Enhanced testing should be implemented in gynecology clinics according to patients’ high risk epidemiological history and clinical symptoms.

The high M/F ratio of reported gonorrhea cases at the Chinese NNDRS may indicate that testing among females is insufficient and likely implies an underestimation of the prevalence of gonorrhea in China. Most female gonorrhea patients are asymptomatic or have similar symptoms as other gynecological diseases, which sets a barrier for finding cases. Therefore, intensifying testing among females will likely provide a more accurately M/F ratio. A program termed “Screen more, Find more” should be advocated by NCSTDC as a national strategy in China to identify more female cases of gonorrhea. In China, the gynecology clinics are coordinated and supervised by the National Center for Women and Children’s Health (NCWCH), China CDC. To strengthen the prevention and control of female gonorrhea, the NCSTDC is therefore initiating more intensive collaborations with NCWCH to enhance the testing for female gonorrhea on a national scale.

In Zhongshan, 80% of the female gonorrhea patients were found in gynecology clinics, which suggested that enhanced testing should be implemented more specifically in the gynecology clinics, especially in asymptomatic females who had high risk behaviours. After interviewing some experienced gynecological physicians, key epidemiological and clinical high-risk indicators for gonorrhea were summarized as follows: 1. Purulent discharge from the cervix or purulent leucorrhea; 2. Symptoms of pelvic inflammatory disease; 3. Positive results of bacterial vaginosis; 4. Increased levels of white blood cells during secretion inspection; 5. Other pain or symptoms in the external genitalia, urethral meatus, vaginal introitus, or perianal region; 6. Multiple sexual partners; 7.Sexual intercourse without prevention measures; 8.Male sexual partner infected with gonorrhea or at high risk of gonorrhea infection.

In US, gonorrhea diagnosis and case reports depend on the NAAT, which has no sensitivity difference between samples collected from males and females. In China, the national guideline recommended NAAT or culture for laboratory diagnosis of female gonorrhea and microscopy, culture and NAAT for male gonorrhea [3]. All of the hospitals in this study are able to perform culture for gonococcus and only four hospitals are able to perform NAATs. Therefore, most (93.5%) of the cases were confirmed by culture. The sensitivity of culture from cervical swabs was not as high as that from urethral male specimens, which may underestimate the positive rate among females and cause an overestimated M/F ratio. In the future, NAAT which display a higher sensitivity and specificity, should be recommended as prior testing method among females. However, culture should be encouraged simultaneously in clinics to collect more isolates for antimicrobial resistance (AMR) surveillance. Currently, only 5% of the isolates in the China gonococcal AMR surveillance program have been collected from females, which results in a biased AMR surveillance [4].

The epidemic of gonorrhea in the USA has been increasing during these past years. In 2018, a total of 583,405 cases of gonorrhea (179.1 cases per 100,000 population) were reported, which is a 5% increase compared with 2017 and an 83% increase compared with the historic low in 2009 [2]. Although the reported cases are much higher than the numbers reported in China, the M/F ratio is only 1.46 and therefore more balanced. According to the US CDC report, 71%–80% of STD cases were found in non-STD clinics [5]. For gonorrhea, US CDC has encouraged physicians who provide primary care to perform STD screening, especially the screening in non-pregnant women by obstetricians-gynecologists. Therefore, strengthening gonorrhea testing and screening in non-STD clinics is very important and the China NCSTDC can learn from the USA experiences.

The findings in this study are subject to at least three limitations. First, the survey was conducted in one city and not on a national scale. The scale of the survey should therefore be expanded in the future. Second, the strategy of enhanced testing of females has not yet been implemented and the effects are currently unknown. Therefore, this public health strategy should be evaluated in more sentinel sites in China. Third, data of genital chlamydial infection was not included in this study. Genital chlamydial infection was a common STD in China. In 2019, the incidence of chlamydial infection was 55.32 cases per 100,000 population [6]. Interestingly, the M/F ratio was only 0.32, which suggested that females are more likely to be tested for genital chlamydial infection rather than gonococcal infection.

## Supporting information

S1 Dataset (XLSX)
